# Refractory *Helicobacter pylori* treatment in resource‐limited settings

**DOI:** 10.1002/jgh3.12934

**Published:** 2023-06-24

**Authors:** Sanjiv Mahadeva

**Affiliations:** ^1^ Division of Gastroenterology, Department of Medicine, Faculty of Medicine University of Malaya Kuala Lumpur Malaysia

Despite a declining prevalence in the last few decades, *Helicobacter pylori* (*H. pylori*) remains a significant cause for upper gastrointestinal diseases globally. Eradicating *H. pylori* facilitates the reduction of gastric cancer,^1^ and it is an effective method of healing peptic ulcer disease.^2^ Testing and treating adult patients with un‐investigated dyspepsia, even in Asia, have been shown to be more cost‐effective than an endoscopy‐based approach.^3^ In Asia, the 1‐week standard triple therapy, comprising a proton pump inhibitor (PPI), amoxicillin, and clarithromycin,^4^ was recommended as first‐line therapy due to its effectiveness, good tolerability, and high patient compliance in the early 21st century.^5^ The first‐line therapy for patients with penicillin allergy would have metronidazole, clarithromycin, and a PPI. However, due to excessive usage of antibiotics globally, an increasing resistance to both metronidazole and clarithromycin has occurred, resulting in a lower success rate of 80% eradication for the standard 1‐week first‐line regime.

The recent Malaysian consensus report on *H. pylori* has recommended increasing the duration of first‐line therapy with amoxicillin, clarithromycin, and a PPI to 2 weeks, with randomized controlled studies in both Malaysia and globally showing an increased *H. pylori* eradication rate in recent times.^6^ An alternative would be to use a high‐dose dual‐therapy regimen for 2 weeks, whereby PPIs are given in multiple doses. The rationale is that increasing intragastric pH to >6 increases *H. pylori* sensitivity to Amoxycillin. A recent study from Malaysia had shown that the eradication for *H. pylori* was 92.8% with a high‐dose dual‐therapy regimen compared with 86.2% with a standard clarithromycin‐based triple‐therapy regimen for 2 weeks.^7^ In regions with a high (>15%) clarithromycin resistance rate, bismuth quadruple therapy, comprising a PPI, bismuth, tetracycline, and metronidazole for a 2‐week duration has been shown to be an effective first‐line therapy.^6^ However, bismuth‐containing compounds are not easily available in the South East Asian region, and hence usually utilized as second‐line therapy.^6^

Despite these well‐proven first‐line eradication regimes, there is still a 10%–15% failure rate. Antibiotic treatment regimens for cases refractory to first‐line therapy should ideally be guided by antibiotic susceptibility testing, to minimize further resistance. *H. pylori* culture based methods to determine antibiotic susceptibility lack precision and are often affected by common use of PPIs and antibiotics. Molecular‐based techniques are the most reliable method to detect *H. pylori* antibiotic resistance currently. However, there is a lack of laboratory facilities for molecular‐based testing in regions with limited healthcare resources.^6^ Furthermore, a multicenter study from Taiwan, which compared molecular resistance guided based antibiotic therapy with empirical therapy based on medication history, demonstrated no significant differences in eradication rates between the two approaches.^8^

In this issue of JGH OPEN, Rattanachaisit et al. report on the efficacy of empirical based therapy for refractory *H. pylori* cases in adults with dyspepsia from Thailand.^9^ Among 1589 *H. pylori* positive dyspeptics, their first‐line regime (predominantly clarithromycin‐based triple therapy) eradication rate was 85.08%. Empirical based second‐line therapy consisted mainly of higher doses of clarithromycin‐based triple therapy, bismuth‐based quadruple therapy, or levofloxacin‐based triple therapy. The eradication rate for empirical based therapy was 69.87%. Twenty six patients received a third‐line regime with a 53.85% eradication rate and 10 patients received a fourth‐line regime with a 50% success rate. The low eradication rates for refractory *H. pylori* cases in this study are believed to be multifactorial. The authors report that their retrospective study did not capture data on treatment adherence, which would have considerably influenced the efficacy of multiple courses of antibiotics. Furthermore, more effective salvage regimens recommended in the guidelines, such as triple therapy with rifabutin, high‐dose dual‐therapy, or furazolidone‐containing regimens,^10^ were not available in their center. Lastly, the re‐treatment with clarithromycin‐based regimes, despite having been used in the first‐line regime, has been shown to lead to a lower eradication rate.^6^

In summary, refractory *H. pylori* infection can be challenging to treat in resource‐limited settings. Although molecular techniques for antibiotic susceptibility testing are ideal, these are not readily available. Most guidelines continue to recommend empirical based treatment regimes, but provided a careful medication history is obtained. Finally, re‐treatment with clarithromycin‐based regimes tends to lead to more resistance and should be avoided.

## References

[1] Ford AC , Yuan Y , Moayyedi P . *Helicobacter pylori* eradication therapy to prevent gastric cancer: systematic review and meta‐analysis. Gut. 2020; 69: 2113–21. 32205420 10.1136/gutjnl-2020-320839

[2] Ford AC , Delaney BC , Forman D , Moayyedi P . Eradication therapy in *Helicobacter pylori* positive peptic ulcer disease: systematic review and economic analysis. Am. J. Gastroenterol. 2004; 99: 1833–55. 15330927 10.1111/j.1572-0241.2004.40014.x

[3] Mahadeva S , Chia YC , Vinothini A , Mohazmi M , Goh KL . Cost‐effectiveness of and satisfaction with a *Helicobacter pylori* “test and treat” strategy compared with prompt endoscopy in young Asians with dyspepsia. Gut. 2008; 57: 1214–20. 18441005 10.1136/gut.2007.147728

[4] Qua CS , Manikam J , Goh KL . Efficacy of 1‐week proton pump inhibitor triple therapy as first‐line *Helicobacter pylori* eradication regime in Asian patients: is it still effective 10 years on? J. Dig. Dis. 2010; 11: 244–8. 20649738 10.1111/j.1751-2980.2010.00445.x

[5] Fock KM , Katelaris P , Sugano K *et al*. Second Asia‐Pacific Consensus Guidelines for *Helicobacter pylori* infection. J. Gastroenterol. Hepatol. 2009; 24: 1587–600. 19788600 10.1111/j.1440-1746.2009.05982.x

[6] Goh KL , Lee YY , Leow AH *et al*. A Malaysian consensus report on the diagnosis and treatment of *Helicobacter pylori* infection. JGH Open. 2023; 7: 261–71. 37125243 10.1002/jgh3.12886 PMC10134769

[7] Hwong‐Ruey Leow A , Chang JV , Goh KL . Searching for an optimal therapy for *H. pylori* eradication: High‐dose proton‐pump inhibitor dual therapy with amoxicillin vs. standard triple therapy for 14 days. Helicobacter. 2020; 25: e12723. 32713104 10.1111/hel.12723

[8] Liou JM , Chen PY , Luo JC *et al*. Efficacies of Genotypic Resistance‐Guided vs Empirical Therapy for Refractory *Helicobacter pylori* Infection. Gastroenterology. 2018; 155: 1109–19. 29964036 10.1053/j.gastro.2018.06.047

[9] Rattanachaisit P , Burana C , Jaroenlapnopparat A *et al*. The prevalence and treatment outcomes of *Helicobacter pylori* infection in a tertiary hospital in Thailand, 2018–2021. JGH Open. 2023; 7: 439–44. 10.1002/jgh3.12916 PMC10290267 37359115

[10] Chey WD , Leontiadis GI , Howden CW , Moss SF . ACG clinical guideline: treatment of *Helicobacter pylori* Infection. Am. J. Gastroenterol. 2017; 112: 212–39. 28071659 10.1038/ajg.2016.563

